# The Effect of Alternating Application of Cold and Hot Compresses on Reduction of Breast Engorgement Among Lactating Mothers

**DOI:** 10.7759/cureus.53134

**Published:** 2024-01-28

**Authors:** Fatimah H Alshakhs, Nouran E Katooa, Hanan A Badr, Hala A Thabet

**Affiliations:** 1 Faculty of Nursing, Maternity and Child Health Department, King Abdulaziz University, Jeddah, SAU; 2 Faculty of Nursing, Women's Health and Midwifery Nursing Department, Mansoura University, Mansoura, EGY

**Keywords:** non-pharmacological methods, compress application, cold gel pack, breast engorgement, nursing care

## Abstract

Background: Breast engorgement is a common issue that affects breastfeeding initiation and continuation. Engorgement can cause exhaustion, making it difficult to care for and feed the infant, and it can cause a mother to wean her baby before she intends to initiate breastfeeding. This study aimed to determine the effectiveness of the alternating application of cold and hot compresses in reducing breast engorgement among lactating mothers.

Design, sample size and setting: A quasi-experimental study design is used to conduct this study. A purposeful sample of 100 lactating mothers was screened in the postnatal ward for the presence of breast engorgement. The 100 mothers with engorgement were divided into two equal groups of 50 mothers each - the first group (the intervention) and the second group (the control) - at the postnatal ward of King Abdulaziz Hospital-National Guard in Alahsa City.

Sampling and tools: The data collection was conducted over five months, from January 2023 until May 2023. Data collection was done through a structured interview questionnaire sheet. The questionnaire was divided into six parts: socio-demographic data, obstetric and initial women assessment, the six-point engorgement scale (SPES), the visual analog scale (VAS), the LATCH breastfeeding charting scale, and the Infant Breastfeeding Assessment Tool (IBFAT).

Result: The present study found no statistically significant difference between the study intervention and control groups in breast engorgement, LATCH level, or overall level of breastfeeding assessment before the intervention. While, in terms of breast engorgement, pain level, attachment, and LATCH, the overall levels of infant breastfeeding assessment across study time had a statistically significant variance among the study and control groups after the intervention at p values=<0.001.

Conclusion and recommendations: The alternating application of cold and hot compresses can have a significant role in reducing breast engorgement among lactating mothers. Women should be encouraged to use hot compresses and cold gel packs as an alternative treatment to reduce engorgement and promote comfort. In addition, the study results can be utilized to aid Saudi Arabian nurses and midwives in understanding the advantages of applying a cold gel pack and a hot compress and to decrease levels of engorgement, improve latch, and relieve discomfort.

## Introduction

Breastfeeding starts after birth, whereas lactation planning starts during pregnancy. The World Health Organization (WHO) has recommended that exclusive breastfeeding start from the baby’s birth until the baby is six months old because colostrum is formed immediately after delivery. Breast milk naturally contains all the vitamins and minerals a child’s body needs to support healthy development. Pregnancy and childbirth cause a number of physiological changes in women’s bodies, including breast growth, darkening of the nipple and areola, and the presence of visible veins on the chest that give off a warm sensation and make the breasts appear fuller [1].

The benefits of breastfeeding for both the mother and her child are both immediate and long-term. There is a substantially lower chance of infants who excessively breastfeed becoming overweight, experiencing high cholesterol, or having high blood pressure as adults. Furthermore, breastfeeding has been associated with lower rates of infant mortality, morbidity, and intestinal disorders, as well as a reduced chance of celiac disease and asthma. Breastfed infants have a stronger connection with their mothers and have better intellectual skills [2].

Breast problems can begin immediately after childbirth or at any moment during lactation. Women experience trouble with breastfeeding at the start of lactation. Breast issues are extremely common in the postpartum period; 40% of breastfeeding mothers experience unpleasant symptoms within the first four days postpartum, and 20% of new mothers, particularly primigravida women, have breast engorgement [3].

Risk factors that could lead to problems with the breasts include insufficient breast emptying, improper nursing techniques, shorter breastfeeding sessions, failure to notice early hunger cues, supplementation of formula, use of a breast milk pump without a medical need, use of pacifiers and traditional nutritional supplements for the infant, and the infant’s poor sucking [4].

Breast engorgement is defined by painful breast swelling brought on by an abrupt increase in milk volume, which makes it challenging for the baby to latch on to the mother's breast. Breast engorgement may occur if a mother's milk production rate exceeds her milk outflow rate or if her infant has difficulty during feeding. Engorgement is brought on by increased lactogenesis, which occurs when prolactin levels rise, and steroid hormone levels decrease among postpartum women [5].

Engorgement results from excessive milk production in the breast and leads to breast fullness. Engorgement can also develop later if a lactating woman skips several feedings and fails to draw enough milk from her breasts. Insufficient nursing or additional factors may make it worse. When the milk ducts are obstructed, the breasts may swell and become tender in a moderate to severe way as they become engorged. Untreated engorgement puts pressure on the milk ducts, frequently resulting in a plugged duct, and it may result in mastitis (breast inflammation). The mother frequently experiences a lump in one breast. The breasts are typically full, hard, tight, tender, painful, and hot to the touch. The temperature may also rise. It causes mastitis, decreased milk supply, cracked nipples, and an early cessation of breastfeeding [6].

Breast engorgement is one of the most prevalent problems among lactating mothers. There are many various pharmacological and non-pharmacological approaches used to control the pain level associated with breast engorgement. Most mothers have a lot of concerns about the pharmacological ways and how they can affect the baby. Breastfeeding mothers intend to choose alternative and complementary therapies because they are worried about the adverse effects of chemical drugs and are searching for the simplest, most direct non-pharmacological methods to treat engorgement [6].

One non-pharmacological technique that can be used to treat breast engorgement is alternate hot and cold compresses. The use of ice is presumed to be calming and to cause vasoconstriction, which reduces blood flow to the skin and minimizes engorgement, whereas the hot compress is presumed to trigger the milk ejection reflex and reduce edema. Because they are more widely available, typically easy to use, convenient, and less expensive than pharmacological techniques, non-pharmacological techniques are becoming more and more popular as acceptable therapy options [6].

Hot compresses are a safe non-pharmacological technique that offers pain relief along with a rapid natural release of milk without side effects and without requiring the use of any medication. The heat can reach deep or superficial layers of the skin. Before feeding, applying hot compresses to the breasts and nipples and expressing milk can reduce discomfort and relax blood vessels, resulting in more blood flow to the breast and nipple [7].

Cold compresses, meanwhile, are used to reduce vascularity and pain and decrease swelling. The first nine to 16 minutes of cold therapy, including cold applications such as ice packs, cold gel packs, and frozen wet towels, initiate a cycle of vasoconstriction where blood flow and local edema are reduced, and lymphatic drainage is improved [8].

These methods are cheap and easy to apply, so mothers can learn how to use them on their own. This study aims to determine the effectiveness of alternating applications of cold gel packs and hot compresses on the reduction of breast engorgement among lactating mothers. The nurse should concentrate on preventing breast engorgement by advising the mother to start breastfeeding as soon as possible after delivery. This gives the infant time to learn to breastfeed even before the breasts become full and firm. Early postpartum treatment is also essential for identifying and treating complications [9].

Significance of the study

Breast engorgement can interfere with the breastfeeding process. Most women complain of breast engorgement after delivery on the third or fourth day postpartum. This problem mostly exists in mothers who had to undergo a cesarean section and is due to delays in the initiation of breastfeeding. Inadequate feeding of the infant leads to engorgement, and the mother also suffers from discomfort, pain, swelling, and other physical and psychological distress [10]. Breast engorgement negatively affects breastfeeding, latching, and sucking. Accordingly, as soon as a breastfeeding issue arises, both mothers and caregivers should recognize it and address it to treat and prevent any complications and progression [10].

Incidence rates of breast engorgement have been reported to range from 20.0% to 77.0% in various studies. Untreated engorgement will result in inadequate milk production and cessation of breastfeeding and, in more severe cases, will develop into inflammation or mastitis. Identifying breast engorgement early conducting appropriate management to maintain mothers’ health and ensuring the correct breastfeeding process is followed are important responsibilities of nurses and midwives [11].

Mothers who experience breast engorgement can be treated using either pharmacological or non-pharmacological techniques. Women are increasingly interested in non-pharmacological techniques such as breast milk expression, cool packs, herbal compresses, acupressure, hot and cold compresses, breast massage, and cold cabbage leaves. Because they are more widely accessible, simple to use, convenient, and affordable than medical interventions, these non-pharmacological techniques are gaining more and more attention as effective treatment options [12].

The primary goal of breast engorgement control is to easily achieve and maintain breast milk flow and to efficiently empty breast milk to prevent engorgement. There is a lack of research in the Kingdom of Saudi Arabia (KSA) about breast engorgement remedies. The current study aims to fill this gap and determine the effectiveness of alternating applications of cold and hot compresses for reducing breast engorgement among lactating mothers.

Aim of the study

This study aims to determine the effectiveness of alternating applications of cold gel packs and hot compresses on the reduction of breast engorgement among lactating mothers.

Research objectives

The objectives of this study are to assess the breast engorgement pre and after alternating application of cold and hot compresses on reduction of breast engorgement among lactating mothers; to determine the effectiveness of the alternating application of cold and hot compresses on the reduction of breast engorgement among lactating mothers; and to find an association between breast engorgement levels pre and after alternating application of cold and hot compresses on reduction of breast engorgement among lactating mothers.

Research hypothesis

H0, the null hypothesis, is that alternating application of cold and hot compresses doesn't reduce breast engorgement. H1, the alternative hypothesis, is that alternating application of cold and hot compresses reduces breast engorgement.

## Materials and methods

Research design and study sample

A quasi-experiment design was used to conduct the study. Purposeful sampling was used in this study. The sample size was calculated using the sample equation and the rule of the sum based on data from related research and the monthly numbers of admissions into the postnatal unit during the data collection period. The total number of mothers has been estimated through the Epi info 7 statistical program based on the following parameters: total population of 400 mothers, expected frequency 50%, margin of errors 5% and confidence level 95%.

The minimum sample size was 200 mothers. The inclusion criteria to recruit the participants are: breast-engorged postnatal mothers with their babies during early postnatal days stayed from three to five days, primiparous or multigravida women who delivered by cesarean section, and gestational age at delivery between 37 and 42 full weeks. The study’s exclusion criteria were mothers who had breast abscesses, mastitis, breast infections, a history of breast surgery, lactating mothers with spontaneous vaginal deliveries (SVD), mothers who had left the hospital by the time of the study, and non-lactating mothers.

Two hundred lactating mothers in the obstetrics ward were screened by the engorgement scale for the presence of breast engorgement, and 100 out of 200 met the inclusion criteria. The 100 mothers who had engorgement were divided into two equal groups (intervention and control); each group was 50 mothers.

The data collection was conducted over five months, from January 2023 until May 2023. The researcher spent four days in the hospital, from 8 a.m. to 2 p.m. The first two months were spent recruiting women who had undergone routine hospital care (the control group), and the other three months were spent recruiting women who participated in the intervention group.

Study setting

The study was carried out at King Abdulaziz Hospital, National Guard, in Al Ahsa city in the Kingdom of Saudi Arabia’s eastern province. The participants were recruited from the obstetrics ward. 

Ethical consideration

The King Abdulaziz University Nursing Faculty’s Ethical Committee in Jeddah provided approval to conduct the study. Next, the King Abdullah International Medical Research Center (KAIMRC) provided permission to access patients (IRB /2794/22). To obtain permission to conduct the study in the postnatal ward and to collect the necessary data while maintaining the confidentiality and privacy of participants’ records and information, an official ethical approval letter was sent to the Director of Nursing Administration at King Abdulaziz Hospital-National Guard. Finally, consent was obtained from the women who met the inclusion criteria before the study was conducted.

Tools for data collection

The tool was developed after a thorough review of the scientific literature, an internet search, and discussions with experts in the obstetric and maternity field. The researcher collected data by using structured questionnaires. The structured questionnaire had six parts:

Part (I): Sociodemographic Data

This part collected sociodemographic data on height, weight, age, level of education, occupation, financial state, place of living, number of children, and body mass index (BMI).

Part (II): Obstetric and Initial Women Assessment

This part dealt with participants’ initial assessment and obstetric history. In this study, the collected data for this part included the number of antenatal visits, breastfeeding education, breast care education, breast problems during pregnancy, clinical examination of the breast by a physician, milk production, discomfort during breastfeeding, immediate skin-to-skin contact, parity, number of postpartum days, initiation of breastfeeding, duration of breastfeeding from each breast, mother position during feeding, and frequency of feeding by hours.

Part (III): Six-Point Engorgement Scale (SPES)

Hills and Humenick created the SPES and Thomas et al. [13] adapted it in the English language. It is a standardized tool with a single inquiry. In this study, it was used to evaluate the severity of breast engorgement on a scale of 1 to 6: 1 for softness and no changes in the breast, 2 for light changes in the breast, 3 for firmness and no tenderness in the breast, 4 for firmness and the beginning of tenderness in the breast, 5 for hardness and tenderness in the breast, and 6 for firmness and tenderness in the breast.

Part (IV): Visual Analog Scale (VAS)

The VAS, adopted from Sung and Wu [14], is used for assessing the level of pain intensity. It consists of blank lines with descriptors of the pain levels placed at either end of the line. In most cases, a 10 centimeter line has been used for measurements. The most frequently used anchoring descriptors are “no pain” and “severe pain.” Postpartum mothers are instructed to point to the image on the line that most accurately represents their level of discomfort. Scores range from 0 (indicating no pain) to 10 (indicating the most severe pain). The VAS is divided into three main sections: 1-3 cm to reflect mild discomfort, 4-7 cm to reflect moderate pain, and 8-10 cm to reflect severe pain.

Part (V): LATCH Breastfeeding Charting Scale

This scale was adopted from El-Hady [9]. The abbreviation LATCH stands for the five essential breastfeeding factors: “L” for how well the infant latches onto the breast, “A” for the frequency of audible swallowing noted, “T” for the mother’s nipple type or condition, “C” for the mother’s level of comfort, and “H” for the amount of assistance the mother requires when holding the baby to the breast. Each of these factors receives a numerical score (0, 1, or 2) according to the scoring system. Additionally, total scores from 0 to 10 are assigned for breastfeeding, with a higher number indicating effective breastfeeding. Last, the evaluation scale for latches is as follows: 1-3, inadequate breastfeeding; 4-6, acceptable breastfeeding; 7-10, satisfactory breastfeeding.

Part (VI): Infant Breastfeeding Assessment Tool (IBFAT)

The IBFAT was adopted from Brugaletta et al. [15]. It is used to assess the natural feeding and behavioral reflexes of healthy newborns. The IBFAT was primarily created to evaluate the efficiency of babies’ breastfeeding practices. It is used to evaluate infant feeding ability from birth to four days after delivery. The infant’s consciousness, rooting reflex, duration of latching, and sucking quality are the four factors that make up the IBFAT. Each of the four factors is given a score ranging from 0 to 3. The higher the score, the more active the newborn’s breastfeeding habit. Evaluation scores range from 0 (unsatisfied) to 3 (very satisfied).

Validation of tools and reliability

Content validity was evaluated by showing the questionnaire to three experts in the maternity, women’s health, and nursing fields. They reviewed the questionnaire for clarity, comprehensiveness, and appropriateness. Essential modifications were made according to these experts’ opinions. The reliability was determined by calculating Cronbach's alpha which was 0.75.

Pilot study

A pilot study was conducted on 10% of the sample (10 participants from the two groups) to test the clarity and testing of the feasibility, simplicity, and applicability of the research process as well as to determine the time allowed to fulfill the developed tool. After the pilot study, no changes were made to the questionnaire. The lactating mothers who were involved in the pilot study were included in the study.

Data collection process

Planning and Assessment Phase

The researcher first prepared the questionnaire. Then, participants were recruited according to the inclusion criteria, and they were divided into two equal groups: the intervention and control groups. Before beginning the data collection, the researcher explained the study's purpose and procedures to each participant and provided her with an informed consent form. Each participant was asked individually about her sociodemographic data.

Implementation Phase

The researcher met with prospective postpartum mothers at the inpatient obstetric ward. The researcher introduced herself to the mothers and gave a brief explanation of the study’s objectives. Mothers who agreed to participate in the study were required to complete an informed consent form. The researcher filled out participants’ questionnaires in both groups.

The mothers in the control group were assessed without implementing any intervention; they received routine care in the hospital which included physical assessment and discharge education. In the intervention group, mothers were assessed before and after applying hot and cold compresses. Both groups’ variables, including sociodemographic data; obstetric and initial assessment data; SPES, VAS, and LATCH scales data; and IBFAT data, were documented in the recording sheet.

The interview and evaluation phase lasted between 20 and 30 minutes for each participant. The questions were asked in simple Arabic, and the researcher recorded the responses. Each participant was given simple written and illustrated instructions on the use of compresses as well as information on goals, outlines, and expected outcomes.

Control group participants who were hospitalized in wards received only routine care and discharge education from hospital nurses. Breast engorgement was evaluated using the checklist for breast engorgement before and after the application of the hot and cold compression. Neither hot nor cold compress was applied.

In the intervention group, before the intervention, an initial assessment of breast engorgement was conducted using a checklist. A cold gel pack (10°C to 18°C, as measured by a lotion thermometer) was placed over the engorged breast [8]. The pack was covered with a clean, dry towel and placed directly inside the mother’s bra. This intervention lasted for 15-20 minutes, and it was replaced every one to two minutes to prevent ischemia.

Thirty minutes after the cold gel pack was taken off, a hot compress (43°C - 46°C, as measured by a lotion thermometer) was placed over the engorged breast [4]. The hot compress was covered with a clean, dry towel as well and was replaced every one to two minutes. This intervention lasted for 15-20 minutes.

After a two-hour interval, the above steps were repeated to ensure the effect of the alternating application. To determine pain levels and engorgement scores, participants were assessed before and after the two rounds of application.

Each participant was given cold and hot compresses when discharged to use at home. Mothers were personally advised on how to use a compress at home and encouraged to apply it twice a day. Mothers were also provided with verbal instructions on how to properly nurse their babies and how to practice good hand hygiene. They were also asked to respond to the baby’s hunger cues by breastfeeding the baby using both breasts every two to three hours for 10-15 minutes. A follow-up was conducted by phone after three days to check whether mothers had experienced pain relief and reduction of engorgement.

Evaluation Phase

In this stage, the levels of breast engorgement, signs and symptoms of engorgement, and engorgement discomfort were assessed using the LATCH scale. The IBFAT, a six-point scale for measuring breast engorgement, and the VAS were used to evaluate the degree of pain. This posttest took about 15-20 minutes for each woman. All the mothers were allowed to ask for clarification on any statement they did not understand.

Data analysis

The collected data were coded, categorized, and tabulated using simple statistics, including frequencies, means, standard deviation, and percentages, as well as an independent sample t-test, Chi-square test, ANOVA test, Student's t-test, paired t-test, and r = correlation coefficient. To identify the relationships among the variables, the Statistical Package for Social Sciences (SPSS) version 24 (IBM Corp., Armonk, NY, USA) was used. Significance was set at p ≤ 0.05 for all tests. Finally, a comparison between the intervention and control groups was done to assess the effectiveness of the application of cold and hot compresses for reducing breast engorgement among lactating mothers.

## Results

The distribution of the studied groups according to their socio-demographic data is shown in Table 1. The table shows that only 14.0% of the study group and the control group were less than 20 years old, while more than one-quarter of the study group and one-third of the control group (28.0% and 34.0%, respectively) were more than 30 and less than 35 years old. On the other hand, nearly one-quarter of the study group and the control group (26% and 24.0%, respectively) were more than 25 to less than 30 years old. In relation to the women's educational level, only 2.0% of the study group and 4.0% of the control group were less than high school, while nearly one-quarter of the study group and more than one-third of the control (20.0% and 34.0%, respectively) had a high school educational level, and the majority of the study and control groups (76.0 and 64.0%, respectively) were graduate or postgraduate (Table 1). 

**Table 1:** Distribution of the studied groups according to their socio-demographic data (n=100) X2: Chi square test, FET: Fisher Exact test * Significant at p ≤ 0.05

Demographic data		Groups				Test of Significance
		Study (n=50)		Control (n=50)		
		No.	%	No.	%	
Age (years)	18-<20	7	14.0	7	14.0	X^2^= 0.474 P= 0.976
	20-<25	10	20.0	9	18.0	
	25-<30	13	26.0	12	24.0	
	30-<35	14	28.0	17	34.0	
	≥35	6	12.0	5	10.0	
Level of education	Illiterate	0	0.0	0	0.0	X^2^= 2.662 P= 0.264
	Elementary	0	0.0	0	0.0	
	Intermediate	2	4.0	1	2.0	
	High school	10	20.0	17	34.0	
	Graduated/ Post graduate	38	76.0	32	64.0	
Occupation	Employee	15	30.0	15	30.0	X^2^= 0.062 P= 0.969
	Housewife	22	44.0	23	46.0	
	Students	13	26.0	12	24.0	
Financial status	Enough	41	82.0	37	74.0	X^2^= 0.932 P= 0.334
	Not enough	9	18.0	13	26.0	
Living place	Urban	20	40.0	27	54.0	FET= 1.967 P= 0.115
	Rural	30	60.0	23	46.0	
Number of children	1	16	32.0	18	36.0	X^2^= 1.287 P= 0.732
	2-3	17	34.0	20	40.0	
	4-5	16	32.0	11	22.0	
	≥6	1	2.0	1	2.0	

Furthermore, nearly half of the study and control groups (44.0% and 46.0%, respectively) were housewives, and nearly one-third (30.0% and 30.0%, respectively) of both groups were employed; on the other hand, nearly one-quarter (26.0% and 24.0% respectively) of both groups were students. Regarding women's financial status, the table illustrates that nearly half (40.0% and 54.0% respectively) of the study and control group were from urban places, furthermore nearly half (60.0% and 46.0% respectively) of the study and control group were from rural places. Regarding the number of children, the table shows that more than one-third (32.0% and 36.0%, respectively) of the study and control groups had only one child, and more than one-third (34.0% and 40.0%, respectively) of the study and control groups had two to three children. Also, nearly one-third (32.0%) of the study group and nearly one-quarter (22.0%) of the control group had four to five children, while only 2.0% of the study and control groups had more than six children. The table also displayed that there was no statistical difference between the study and control groups in relation to all socio-demographic data (p > 0.05) (Table 1). 

Table 2 below reveals the studied groups according to their weight, height, and BMI. Concerning the mother's weight, the table displayed that the mean weights of the study and control groups were 65.32±9.013 and 62.92±8.068, respectively. With respect to height, the mean heights of both groups were 160.76±5.093 and 160.86±6.260, respectively. In relation to the BMI, more than half (56.0% and 50.0%, respectively) of both groups were overweight. The table also showed that the mean BMI of both groups was 25.25±3.034 and 24.37±3.206. In the comparison between the study and control group, it was revealed that there were no statistically significant differences in weight, height, or BMI (all p > 0.05) (Table 2). 

**Table 2:** Distribution of the studied groups according to their weight, height and BMI (n=100) X2: Chi square test, t: Student's t test * Significant at p ≤0.05

Items		Groups				Test of Significance
		Study (n=50)		Control (n=50)		
		No.	%	No.	%	
Weight (kg)	Min-Max	49.0-91.0		48.0-83.0		t= 1.968 P= 0.164
	Mean± SD	65.32± 9.013		62.92± 8.068		
Height (cm)	Min-Max	148.0-172.0		148.0-173.0		t= 0.008 P= 0.930
	Mean± SD	160.76± 5.093		160.86± 6.260		
BMI	Underweight	0	0.0	1	2.0	X^2^= 1.589 P= 0.662
	Normal weight	19	38.0	22	44.0	
	Overweight	28	56.0	25	50.0	
	Obese	3	6.0	2	4.0	
	Min-Max	19.81-33.25		17.21-31.20		t= 1.964 P= 0.164
	Mean± SD	25.25± 3.034		24.37± 3.206		

Regarding the number of antenatal visits, nearly two-thirds (60.0%) of the study group had three to four visits, compared to more than one-third (38.0%) of the control group. Furthermore, more than one-third (38.0%) of the study group had more than five visits, compared to more than half (52.0%) of the control group. The table also shows that more than half (54.0% and 58.0%, respectively) of both groups did not receive breastfeeding education or breast care education. Furthermore, more than half (50.0% and 58.0%, respectively) of both groups have breast problems during pregnancy, and flat nipples were stated as the most common problem by the majority (92.0% and 86.0%, respectively) of both groups (Table 3).

**Table 3:** Distribution of the studied groups according to their obstetrical and initial mother’s assessment (n=100) X2: Chi square test, FET: Fisher exact test, NA: Not applicable * Significant at p ≤0.05

Obstetrical and initial mother’s assessment		Groups				Test of Significance
		Study (n=50)		Control (n=50)		
		No.	%	No.	%	
Number of antenatal visits	1-2	1	2.0	5	10.0	X^2^= 6.225 P= 0.044*
	3-4	30	60.0	19	38.0	
	≥5	19	38.0	26	52.0	
Breastfeeding education	No	27	54.0	29	58.0	FET= 0.162 P= 0.420
	Yes	23	46.0	21	42.0	
Breast care education	No	27	54.0	29	58.0	FET= 0.162 P= 0.420
	Yes	23	46.0	21	42.0	
Breast problems during pregnancy	No	25	50.0	21	42.0	X^2^= 0.644 P= 0.422
	Yes	25	50.0	29	58.0	
	Flat nipple	23	92.0	25	86.2	X^2^= 0.456 P= 0.499
	Inverted nipple	2	8.0	4	13.8	
Breast examination by a physician	No	36	72.0	40	80.0	X^2^= 0.877 P= 0.349
	Yes	14	28.0	10	20.0	
Milk production	No	8	16.0	25	50.0	X^2^= 13.071 P= 0.000*
	Yes	42	84.0	25	50.0	
Discomfort during breastfeeding	No	2	4.0	12	24.0	X^2^= 8.306 P= 0.004*
	Yes	48	96.0	38	76.0	
Immediate skin-to-skin contact	No	22	44.0	22	44.0	X^2^= NA
	Yes	28	56.0	28	56.0	
Parity	Primi para	16	32.0	18	36.0	X^2^= 1.223 P= 0.543
	Secondary Para	17	34.0	12	24.0	
	Multi Para	17	34.0	20	40.0	
Number of post-partum days	Three	13	26.0	11	22.0	X^2^= 0.693 P= 0.707
	Four	20	40.0	18	36.0	
	Five and more	17	34.0	21	42.0	
Initiation of breast feeding	Within 8 hours	7	14.0	16	32.0	X^2^= 5.108 P= 0.164
	9-12 hours	15	30.0	14	28.0	
	12 hours	20	40.0	13	26.0	
	1 day	8	16.0	7	14.0	
Duration of feeding during each feed from the breast	<10 min	2	4.0	7	14.0	X^2^= 3.087 P= 0.378
	10-20 min	33	66.0	30	60.0	
	20-30 min	13	26.0	11	22.0	
	≥30 min	2	4.0	2	4.0	
Mother’s position during breast feeding	Sitting	30	60.0	30	60.0	X^2^= NA
	Side-lying	20	40.0	20	40.0	
Frequency of feeding per hour	1 hour	4	8.0	4	8.0	X^2^= 0.050 P= 0.975
	2 hours	32	64.0	31	62.0	
	3 hours	14	28.0	15	30.0	
Bottle feeding	No	19	38.0	32	64.0	X^2^= 6.763 P= 0.009*
	Yes	31	62.0	18	36.0	

The table also shows that the majority (72.0% and 80.0%, respectively) of the study and control groups did not have breast examinations performed by physicians. The majority (80.0%) of the study group and half of the control group have milk production in their breasts. Furthermore, the majority (96.0% and 76.0%, respectively) of the study and control groups had discomfort during breastfeeding. Similarly, more than half (56.0% and 56.0%, respectively) of the study and control groups had immediate skin-to-skin contact. In relation to parity, the table shows that nearly one-third (32.0% and 36.0% respectively) of both groups were primi-para, more than one-third (34.0%) of the study group and nearly one-quarter (24.0%) of the control group were secondary para, and more than one-third (34.0% and 40.0%, respectively) of both groups were multi-para (Table 3).

In relation to the number of postpartum days, more than one-third (40.0% and 36.0%, respectively) of the study and control group were four days postpartum, and more than one-third (34.0% and 42.0%, respectively) of the study and control group were five and more. Only 14.0% of the study group, as compared to nearly one-third (32.0%) of the control group, initiated breastfeeding within eight hours, while nearly half (40.0%) of the study group, as compared to more than one-quarter (26.0%) of the control group, initiated it after 12 hours (Table 3).

As for the duration of feeding during each feed from the breast, the table shows that a great proportion (66.0% and 60.0%, respectively) of both groups fed their infants for 10-20 minutes using the sitting position among more than half (60.0% and 60.0%, respectively) of both groups, with a two-hour frequency among more than half (64.0% and 62.0%, respectively) of them. The data also revealed that more than half (62.0%) of the study group, as compared to more than one-third (36.0%) of the control group, used bottle feeding (Table 3).

In the comparison between the study and control group, it was revealed that there were no statistically significant differences in all maternal obstetrical data except for data related to the number of antenatal visits, discomfort during breastfeeding, and bottle feeding (p = 0.044, p = 0.000, and p = 0.009, respectively) (Table 3).

It was identified that nearly half (42.0%) of the study group and nearly one-third (30.0%) of the control group had firm or beginning of tenderness in breast pre-intervention, with no statistically significant difference between the two groups before the intervention (X2 = 9.050, P = 0.060). While after the intervention, none of the study groups, as compared to 14.0% of the control group, had firm or beginning tenderness in the breast, there was a statistically significant difference between the two groups after the intervention (X2 = 14.886, P = 0.005) (Table 4).

**Table 4:** Distribution of studied groups according to their levels of breast engorgement before and after the intervention (n=100) X2: Chi square test, the difference b/w the study and control group * Statistically significant at P ≤0.05

Time of assessment	Levels	Groups				test of significance
		Study (n=50)		Control (n=50)		
		No.	%	No.	%	
Pre-intervention	Soft/no changes in breast	0	0.0	0	0.0	X^2^= 9.050 P= 0.060
	Light changes in the breast	2	4.0	11	22.0	
	Firm/non-tender breast	17	34.0	11	22.0	
	Firm/beginning of tenderness	21	42.0	15	30.0	
	Firm/tender breast	9	18.0	11	22.0	
	Very firm/very tender breast	1	2.0	2	4.0	
After intervention	Soft/no changes in breast	11	22.0	4	8.0	X^2^= 14.886 P= 0.005*
	Light changes in the breast	28	56.0	20	40.0	
	Firm/non-tender breast	11	22.0	17	34.0	
	Firm/beginning of tenderness	0	0.0	7	14.0	
	Firm/tender breast	0	0.0	2	4.0	
	Very firm/very tender breast	0	0.0	0	0.0	

It was observed that none of the study groups, as compared to 14.0% of the control group, had any pain pre-intervention, with a statistically significant difference between the two groups before the intervention (X2 = 9.097, P = 0.028), while after the intervention, the majority (70.0%) of the study group, as compared to only 24.0% of the control group, had firm no pain, with a statistically significant difference between the two groups after the intervention (X2 = 21.389, P = 0.000) (Table 5).

**Table 5:** Distribution of studied groups according to levels of LATCH before and after intervention (n=100) X2: Chi square test, the difference b/w the study and control group * Statistically significant at P ≤0.05

Time of assessment	Levels	Groups					test of significance
		Study (n=50)		Control (n=50)			
		No.	%		No.	%	
Pre-intervention	No	0	0.0		7	14.0	X^2^= 9.097 P= 0.028*
	Mild	18	36.0		21	42.0	
	Moderate	28	56.0		19	38.0	
	Sever	4	8.0		3	6.0	
After intervention	No	35	70.0		12	24.0	X^2^= 21.389 P= 0.000*
	Mild	14	28.0		34	68.0	
	Moderate	1	2.0		4	8.0	
	Severe	0	0.0		0	0.0	

It was observed before the intervention that there were no statistically significant differences between the study and control groups in relation to the type of nipple, comfort, and amount of help latching on items (X2 = 3.032, P = 0.220, X2 = 5.494, P = 0.064, and X2 = 4.239, P = 0.120, respectively), but there were statistically significant differences between the study and control group in relation to latching onto breasts and audible swallowing items (X2 = 6.132, P = 0.047*, and X2 = 16.538, P = 0.000*, respectively). After the intervention, there was a statistically significant difference between the study and control groups in relation to all levels of latch-on items (P ≤ 0.05) (Table 6).

**Table 6:** Distribution of studied groups according to levels of pain before and after intervention (n=100) X2: Chi square test, the difference b/w the study and control group

Time of assessment	Levels		Groups				test of significance
			Study (n=50)		Control (n=50)		
			No.	%	No.	%	
Pre-intervention	How to latch into the breast	Too sleepy	8	16.0	18	36.0	X^2^= 6.132 P= 0.047*
		Repeated attempts	30	60.0	26	52.0	
		Grasp breast	12	24.0	6	12.0	
	Audible swallowing	None	2	4.0	17	34.0	X^2^= 16.538 P= 0.000*
		A few with simulation	41	82.0	24	48.0	
		Spontaneous	7	14.0	9	18.0	
	Type of nipple	Inverted	3	6.0	3	6.0	X^2^= 3.032 P= 0.220
		Flat	35	70.0	27	54.0	
		Everted	12	24.0	20	40.0	
	Comfort	Engorged	5	10.0	7	14.0	X^2^= 5.494 P= 0.064
		Filling, tendered	37	74.0	26	52.0	
		Soft/non-tender	8	16.0	17	34.0	
	Amount of help	Need help	9	18.0	17	34.0	X^2^= 4.239 P= 0.120
		Minimal assist	19	38.0	19	38.0	
		No assist	22	44.0	14	28.0	
After intervention	How to latch into the breast	Too sleepy	2	4.0	5	10.0	X^2^= 27.445 P= 0.000*
		Repeated attempts	13	26.0	36	72.0	
		Grasp breast	35	70.0	9	18.0	
	Audible swallowing	None	0	0.0	2	4.0	X^2^= 7.054 P= 0.029*
		A few with simulation	21	42.0	31	62.0	
		Spontaneous	29	58.0	17	34.0	
	Type of nipple	Inverted	0	0.0	0	0.0	X^2^= 13.255 P= 0.000*
		Flat	1	2.0	14	28.0	
		Everted	49	98.0	36	72.0	
	Comfort	Engorged	0	0.0	4	8.0	X^2^= 21.336 P= 0.000*
		Filling, tendered	8	16.0	26	52.0	
		Soft/non-tender	42	84.0	20	40.0	
	Amount of help	Need help	0	0.0	4	8.0	X^2^= 6.043 P= 0.049*
		Minimal assist	14	28.0	19	38.0	
		No assist	36	72.0	27	54.0	

It was observed before the intervention that there was no statistically significant difference between the study and control groups in relation to total levels of LATCH over study time (X2 = 3.328, P = 0.189). After the intervention, there was a statistically significant difference between the study and control groups in relation to total levels of LATCH over study time (X2 = 9.514, P = 0.009*) (Table 7).

**Table 7:** Distribution of studied groups according to total levels of LATCH before and after intervention (n=100) X2: Chi square test, the difference b/w the study and control group * Statistically significant at P ≤0.05

Time of assessment	Levels	Groups				test of significance
		Study (n=50)		Control (n=50)		
		No.	%	No.	%	
Pre-intervention	Inadequate breastfeeding	6	12.0	12	24.0	X^2^= 3.328 P= 0.189
	Acceptable breastfeeding	31	62.0	23	46.0	
	Satisfactory breastfeeding	13	26.0	15	30.0	
After intervention	Inadequate breastfeeding	0	0.0	2	4.0	X^2^= 9.514 P= 0.009*
	Acceptable breastfeeding	6	12.0	17	34.0	
	Satisfactory breastfeeding	44	88.0	31	62.0	

It was observed that only 6.0% of the study group, as compared to 16.0% of the control group, had infants that had no effort needed in arousal, with a statistically significant difference between the two groups before the intervention (X2 = 15.406, P = 0.002*). After the intervention, the majority (86.0%) of the study group, as compared to nearly one-third (34.0%) of the control group, had infants that had no effort needed in arousal, with a statistically significant difference between the two groups after the intervention (X2 = 17.614, P = 0.000*). Regarding infant rooting, it was observed that nearly one-third (32.0%) of the study group, as compared to nearly one-quarter (24.0%) of the control group, had infants that rooted effectively, with a statistically significant difference between the two groups before the intervention (X2 = 10.857, P = 0.013*) (Table 8).

**Table 8:** Distribution of studied groups according to levels of infant breastfeeding assessment before and after intervention (n=100) X2: Chi square test, the difference b/w the study and control group * Statistically significant at P ≤0.05

Time of assessment	Levels		Groups				test of significance
			Study (n=50)		Control (n=50)		
			No.	%	No.	%	
Pre-intervention	Baby arousal	Could not arouse	0	0.0	2	4.0	X^2^= 15.406 P= 0.002*
		Unbundled baby	4	8.0	15	30.0	
		Mild simulation	43	86.0	25	50.0	
		No effort needed	3	6.0	8	16.0	
	Rooting	Did not root	0	0.0	2	4.0	X^2^= 10.857 P= 0.013*
		Rooted poorly	2	4.0	12	24.0	
		Need coaxing	32	64.0	24	48.0	
		Rooted effectively	16	32.0	12	24.0	
	Duration b/w latch and suck	Did not feed	0	0.0	0	0.0	X^2^= 3.220 P= 0.200
		Over 10 min	0	0.0	3	6.0	
		3-10 min	22	44.0	19	38.0	
		0-3 min	28	56.0	28	56.0	
	Sucking pattern	Did not suck	0	0.0	4	8.0	X^2^= 12.051 P= 0.007*
		Sucked poorly	5	10.0	12	24.0	
		Sucked on & off	43	86.0	28	56.0	
		Sucked well	2	4.0	6	12.0	
After intervention	Baby arousal	Could not arouse	0	0.0	0	0.0	X^2^= 17.614 P= 0.000*
		Unbundled baby	0	0.0	11	22.0	
		Mild simulation	16	32.0	22	44.0	
		No effort needed	34	68.0	17	34.0	
	Rooting	Did not root	0	0.0	1	2.0	X^2^= 16.838 P= 0.001*
		Rooted poorly	1	2.0	8	16.0	
		Need coaxing	7	14.0	18	36.0	
		Rooted effectively	42	84.0	23	46.0	
	Duration b/w latch and suck	Did not feed	0	0.0	0	0.0	X^2^= 5.556 P= 0.062
		Over 10 min	0	0.0	1	2.0	
		3-10 min	5	10.0	13	26.0	
		0-3 min	45	90.0	36	72.0	
	Sucking pattern	Did not suck	0	0.0	0	0.0	X^2^= 11.244 P= 0.004*
		Sucked poorly	0	0.0	7	14.0	
		Sucked on & off	25	50.0	30	60.0	
		Sucked well	25	50.0	13	26.0	

After the intervention, the majority (84.0%) of the study group, as compared to more than one-third (46.0%) of the control group, had infants that rooted effectively, with a statistically significant difference between the two groups after the intervention (X2 = 16.838, P = 0.001*). In relation to the duration between latch and sucking, the data revealed that nearly half (56.0%) of both groups had infants with a zero-to-three-minute duration, with no statistically significant difference between the two groups before the intervention (X2 = 3.220, P = 0.200) (Table 8).

After the intervention, the majority (90.0%) of the study group, as compared to less than three-quarters (72.0%) of the control group, had infants with zero-to-three-minute duration, with no statistically significant difference between the two groups after the intervention (X2 = 5.556, P = 0.062). As for the sucking pattern, it was observed that only 4.0% of the study group, as compared to 12.0% of the control group, had infants that sucked well, with a statistically significant difference between the two groups before the intervention (X2 = 12.051, P = 0.007*). While after the intervention, half of the study group, as compared to approximately one-quarter (26.0%) of the control group, had infants that sucked well, with a statistically significant difference between the two groups after the intervention (X2 = 11.244, P = 0.004*) (Table 8).

It was observed before the intervention that there was no statistically significant difference between the study and control groups in relation to the total levels of infant breastfeeding assessment over study time (X2 = 5.543, P = 0.063). After the intervention, there was a statistically significant difference between the study and control groups in relation to the total levels of infant breastfeeding assessment over study time (X2 = 14.600, P = 0.001*) (Table 9).

**Table 9:** Distribution of studied groups according to total levels of infant breastfeeding assessment before and after intervention (n=100) X2: Chi square test, the difference b/w the study and control group * Statistically significant at P ≤0.05

Time of assessment	Levels	Groups				test of significance
		Study (n=50)		Control (n=50)		
		No.	%	No.	%	
Pre-intervention	Poor	0	0.0	4	8.0	X^2^= 5.543 P= 0.063
	Fair	18	36.0	22	44.0	
	Good	32	64.0	24	48.0	
After intervention	Poor	0	0.0	1	2.0	X^2^= 14.600 P= 0.001*
	Fair	1	2.0	14	28.0	
	Good	49	98.0	35	70.0	
test of significance		X^2b^= 18.778 P= 0.000*		X^2c^= 5.629 P= 0.059		

It was observed that nearly half (46.0%) of mothers in the study group, as compared to only 8.0% of the control group, were very pleased with breastfeeding, with a statistically significant difference between the study and control groups in relation to the mothers’ evaluation of the breastfeeding (X2 = 32.299, P = 0.000*) (Table 10). 

**Table 10:** Distribution of studied groups according to the mothers’ evaluation of the breastfeeding (n=100) X2: Chi square test, the difference b/w the study and control group * Statistically significant at P ≤0.05

Item		Groups				Test of Significance
		Study (n=50)		Control (n=50)		
		No.	%	No.	%	
Mothers’ evaluation of the breastfeeding	Not pleased	0	0.0	6	12.0	X^2^= 32.299 P= 0.000*
	Fairly pleased	3	6.0	20	40.0	
	Pleased	24	48.0	20	40.0	
	Very pleased	23	46.0	4	8.0	

In relation to breast engorgement, it was observed that there was no significant difference between the study and control group before the intervention (X2a = 9.050, P = 0.060), while there was a significant difference between the study and control group after the intervention (X2b = 14.886, P = 0.005*). As for pain, it was observed that there was a significant difference between the study and control groups before and after the intervention (X2a = 9.097, P = 0.028*, and X2b = 21.389, P = 0.000*). Regarding LATCH, it was observed that there was no significant difference between the study and control groups before the intervention (X2a = 3.328, P = 0.189), while there was a significant difference between the study and control groups after the intervention (X2b = 9.514, P = 0.009*) (Table 11).

**Table 11:** Distribution of studied groups according to their levels of breast engorgement, pain, and LATCH before and after intervention (n=100) X2: Chi square test X2a: The difference b/w the study and control group before the intervention X2b: The difference b/w the study and control group after the intervention * Statistically significant at P ≤0.05

Item of assessment	Levels	Pre-intervention				After intervention				Test of significance
		Study (n=50)		Control (n=50)		Study (n=50)		Control (n=50)		
		No.	%	No.	%	No.	%	No.	%	
Breast engorgement	Soft/no changes in breast	0	0.0	0	0.0	11	22.0	4	8.0	X^2a^= 9.050 P= 0.060 X^2b^= 14.886 P= 0.005*
	Light changes in the breast	2	4.0	11	22.0	28	56.0	20	40.0	
	Firm/non-tender breast	17	34.0	11	22.0	11	22.0	17	34.0	
	Firm/beginning of tenderness	21	42.0	15	30.0	0	0.0	7	14.0	
	Firm/tender breast	9	18.0	11	22.0	0	0.0	2	4.0	
	Very firm/very tender breast	1	2.0	2	4.0	0	0.0	0	0.0	
Pain	No	0	0.0	7	14.0	35	70.0	12	24.0	X^2a^= 9.097 P= 0.028* X^2b^= 21.389 P= 0.000*
	Mild	18	36.0	21	42.0	14	28.0	34	68.0	
	Moderate	28	56.0	19	38.0	1	2.0	4	8.0	
	Sever	4	8.0	3	6.0	0	0.0	0	0.0	
LATCH	Inadequate breastfeeding	6	12.0	12	24.0	0	0.0	2	4.0	X^2a^= 3.328 P= 0.189 X^2b^= 9.514 P= 0.009*
	Acceptable breastfeeding	31	62.0	23	46.0	6	12.0	17	34.0	
	Satisfactory breastfeeding	13	26.0	15	30.0	44	88.0	31	62.0	

There was no significant relation between all participants' obstetrical data and their breast engorgement except in the study group on the number of antenatal visits, the frequency of feeding per hour before the intervention, the mother's position during breastfeeding, and the frequency of feeding per hour before the intervention and in the mother’s position during breastfeeding and the frequency of feeding per hour after the intervention. Also, in the control group, the significant relation was present only in discomfort during breastfeeding, the number of postpartum days before the intervention, and the number of postpartum days after the intervention (Table 12).

**Table 12:** Relationship between the studied group's obstetrical and initial mother’s assessment and their breast engorgement (n=100) X2: Chi square test, the difference b/w the study and control group * Statistically significant at P ≤0.05

Obstetrical and initial mother’s assessment	Breast Engorgement			
	Study group (n=50)		Control group (n=50)	
	Pre-intervention	Post-intervention	Pre-intervention	Post-intervention
Number of antenatal visits	X2= 26.858 P= 0.001*	X2= 0.900 P= 0.925	X2= 3.979 P= 0.859	X^2^= 7.706 P= 0.463
Breastfeeding education	X2= 2.655 P= 0.617	X2= 1.900 P= 0.387	X2= 6.610 P= 0.158	X^2^= 0.608 P= 0.962
Breast care education	X2= 2.655 P= 0.617	X2= 1.900 P= 0.387	X2= 6.610 P= 0.158	X^2^= 0.608 P= 0.962
Breast problems during pregnancy	X2= 1.598 P= 0.809	X2= 1.052 P= 0.591	X2= 8.849 P= 0.065	X^2^= 8.154 P= 0.086
Breast examination by a physician	X2= 8.715 P= 0.069	X2= 0.510 P= 0.775	X2= 7.633 P= 0.106	X^2^= 5.793 P= 0.215
Milk production	X2= 6.740 P= 0.148	X2= 5.550 P= 0.062	X2= 3.600 P= 0.463	X^2^= 1.472 P= 0.832
Discomfort during breastfeeding	X2= 2.342 P= 0.673	X2= 2.652 P= 0.266	X2= 9.995 P= 0.041*	X^2^= 8.488 P= 0.075
Immediate skin-to-skin contact	X^2^= 4.271 P= 0.371	X2= 2.985 P= 0.225	X2= 4.779 P= 0.311	X^2^= 6.924 P= 0.140
Parity	X^2^= 11.996 P= 0.151	X2= 5.863 P= 0.210	X2= 13.041 P= 0.110	X^2^= 12.952 P= 0.113
Number of post-partum days	X^2^= 14.802 P= 0.063	X2= 3.250 P= 0.517	X2= 21.647 P= 0.006*	X^2^= 18.664 P= 0.017*
Initiation of breastfeeding	X^2^= 12.560 P= 0.402	X2= 3.088 P= 0.798	X2= 20.338 P= 0.061	X^2^= 9.846 P= 0.629
Duration of feeding during each feed from the breast	X^2^= 6.824 P= 0.145	X2= 9.419 P= 0.151	X2= 16.165 P= 0.184	X^2^= 11.018 P= 0.527
Mother’s position during breastfeeding	X2= 14.206 P= 0.288	X2= 6.588 P= 0.037*	X2= 4.899 P= 0.298	X^2^= 1.192 P= 0.879
Frequency of feeding per hour	X2= 16.820 P= 0.032*	X2= 11.772 P= 0.019*	X2= 6.314 P= 0.612	X^2^= 3.905 P= 0.866
Bottle feeding	X2= 7.864 P= 0.097	X2= 1.877 P= 0.391	X2= 7.123 P= 0.130	X^2^= 9.805 P= 0.044*

There was no significant relation between all participants' obstetrical and initial mother’s assessment and their level of pain except in the study group on discomfort during breastfeeding and immediate skin-to-skin contact before the intervention and in breast problems during pregnancy after the intervention. Also, in the control group, the significant relation was present only in breast problems during pregnancy, discomfort during breastfeeding, mother’s position during breastfeeding, and bottle feeding before the intervention and the number of postpartum days, the discomfort during breastfeeding, parity, and frequency of feeding per hour after the intervention (Table 13).

**Table 13:** Relationship between the studied groups' obstetrical and initial mother’s assessment and their level of pain (n=100) X2: Chi square test, the difference b/w the study and control group NA: Not applicable * Statistically significant at P ≤0.05

Obstetrical and initial mother’s assessment	level of pain			
	Study group (n=50)		Control group (n=50)	
	Pre-intervention	Post-intervention	Pre-intervention	Post intervention
Number of antenatal visits	X2= 2.183 P= 0.702	X^2^= 6.632 P= 0.157	X^2^= 2.790 P= 0.835	X^2^= 1.908 P= 0.753
Breastfeeding education	X2= 3.272 P= 0.195	X^2^= 1.863 P= 0.394	X^2^= 1.274 P= 0.735	X^2^= 2.731 P= 0.255
Breast care education	X2= 3.272 P= 0.195	X^2^= 1.863 P= 0.394	X^2^= 1.274 P= 0.735	X^2^= 2.731 P= 0.255
Breast problems during pregnancy	X2= NA	X^2^= 10.457 P= 0.005*	X^2^= 9.854 P= 0.019*	X^2^= 4.220 P= 0.121
Breast examination by a physician	X2= 1.086 P= 0.581	X^2^= 4.082 P= 0.130	X^2^= 6.736 P= 0.081	X^2^= 0.153 P= 0.926
Milk production	X2= 5.357 P= 0.069	X^2^= 0.574 P= 0.751	X^2^= 5.148 P= 0.161	X^2^= 1.804 P= 0.406
Discomfort during breastfeeding	X2= 5.874 P= 0.053*	X^2^= 5.357 P= 0.069	X^2^= 13.478 P= 0.004*	X^2^= 10.628 P= 0.005*
Immediate skin-to-skin contact	X2= 7.225 P= 0.027*	X^2^= 1.994 P= 0.369	X^2^= 1.014 P= 0.798	X^2^= 5.695 P= 0.058
Parity	X2= 6.802 P= 0.147	X^2^= 5.334 P= 0.255	X^2^= 10.842 P= 0.093	X^2^= 11.776 P= 0.019*
Number of post-partum days	X2= 3.419 P= 0.490	X^2^= 6.821 P= 0.146	X^2^= 12.038 P= 0.061	X^2^= 5.437 P= 0.245
Initiation of breastfeeding	X2= 1.933 P= 0.926	X^2^= 3.560 P= 0.736	X^2^= 12.465 P= 0.188	X^2^= 10.287 P= 0.113
Duration of feeding during each feed from the breast	X2= 7.022 P= 0.319	X^2^= 9.950 P= 0.127	X^2^= 6.037 P= 0.736	X^2^= 3.189 P= 0.785
Mother’s position during breastfeeding	X2= 0.529 P= 0.768	X^2^= 0.714 P= 0.700	X^2^= 13.450 P= 0.004*	X^2^= 5.208 P= 0.074
Frequency of feeding per hour	X2= 5.689 P= 0.224	X^2^= 2.388 P= 0.665	X^2^= 9.009 P= 0.173	X^2^= 9.639 P= 0.047*
Bottle feeding	X2= 0.969 P= 0.616	X^2^= 2.219 P= 0.330	X^2^= 9.186 P= 0.027*	X^2^= 1.044 P= 0.593

There was no significant relation between all participants' obstetrical data and their level of LATCH except in the study group, on breast examination by physician, milk production, immediate skin-to-skin contact, and duration of feeding during each feed from breast before the intervention and in breast examination by physician after the intervention, also in the control group the significant relation present only in breast problems during pregnancy, breast examination by physician, discomfort during breastfeeding, parity, and number of post-partum days before the intervention and breast problems during pregnancy, and number of post- partum days after the intervention (Table 14).

**Table 14:** Relationship between the studied groups' obstetrical and initial mother’s assessment and their level of LATCH (N=100) X2: Chi square test, the difference b/w the study and control group NA: Not applicable * Statistically significant at P ≤0.05

Obstetrical and initial mother’s assessment	Level of LATCH			
	Study		Control	
	Pre-intervention	Post-intervention	Pre-intervention	Post-intervention
Number of antenatal visits	X2= 1.006 P= 0.909	X^2^= 1.572 P= 0.456	X^2^= 7.114 P= 0.130	X^2^= 3.915 P= 0.418
Breastfeeding education	X2= 4.589 P= 0.101	X^2^= 1.172 P= 0.279	X^2^= 2.783 P= 0.249	X^2^= 4.805 P= 0.090
Breast care education	X2= 4.589 P= 0.101	X^2^= 1.172 P= 0.279	X^2^= 2.783 P= 0.249	X^2^= 4.805 P= 0.090
Breast problems during pregnancy	X2= 4.622 P= 0.099	X^2^= 3.030 P= 0.082	X^2^= 14.967 P= 0.001*	X^2^= 8.871 P= 0.012*
Breast examination by a physician	X2= 10.418 P= 0.005*	X^2^= 5.057 P= 0.025*	X^2^= 10.418 P= 0.005*	X^2^= 3.961 P= 0.138
Milk production	X2= 6.086 P= 0.048*	X^2^= 0.002 P= 0.962	X^2^= 2.020 P= 0.364	X^2^= 0.091 P= 0.955
Discomfort during breastfeeding	X2= 0.760 P= 0.684	X^2^= 0.284 P= 0.594	X^2^= 6.391 P= 0.041*	X^2^= 3.186 P= 0.203
Immediate skin-to-skin contact	X2= 6.300 P= 0.043*	X^2^= 0.100 P= 0.752	X^2^= 1.343 P= 0.511	X^2^= 0.933 P= 0.627
Parity	X2= 6.107 P= 0.191	X^2^= 1.294 P= 0.524	X^2^= 12.274 P= 0.015*	X^2^= 6.936 P= 0.139
Number of post-partum days	X2= 7.916 P= 0.095	X^2^= 3.988 P= 0.136	X^2^= 14.059 P= 0.007*	X^2^= 9.748 P= 0.045*
Initiation of breastfeeding	X2= 5.525 P= 0.478	X^2^= 1.941 P= 0.585	X^2^= 5.198 P= 0.519	X^2^= 4.718 P= 0.580
Duration of feeding during each feed from the breast	X2= 20.030 P= 0.003*	X^2^= 5.091 P= 0.165	X^2^= 4.025 P= 0.673	X^2^= 4.484 P= 0.611
Mother’s position during breastfeeding	X2= 4.921 P= 0.085	X^2^= 1.547 P= 0.214	X^2^= 0.507 P= 0.776	X^2^= 1.834 P= 0.400
Frequency of feeding per hour	X2= 2.937 P= 0.568	X^2^= 5.188 P= 0.075	X^2^= 8.331 P= 0.080	X^2^= 20.829 P= 0.000*
Bottle feeding	X2= 0.506 P= 0.777	X^2^= 0.063 P= 0.802	X^2^= 3.625 P= 0.163	X^2^= 1.726 P= 0.422

## Discussion

The “gold standard” dietary source during the first several months after birth is unquestionably breastfeeding. Although being a mother is a wonderful and special experience, a variety of medical, psychological, and social issues can frequently occur in the postpartum period, and these issues can affect breastfeeding. One of the most frequent mild discomforts experienced by nursing mothers, especially primiparous mothers, is breast engorgement.‏‏‏‏

Breastfeeding issues, insufficient milk production, and a decreased propensity to breastfeed can all result from engorgement and the accompanying pain. A poor latch is the most typical sign of breastfeeding-related pain. To address breastfeeding issues, numerous pharmacological and non-pharmacological approaches have been suggested. The former has a variety of adverse effects, and mothers are often concerned about how these approaches will affect their unborn children. Thus, non-pharmacological approaches have attracted a great deal of interest lately [16]. ‏ ‏

Numerous nonpharmacological approaches exist, including peppermint and ginger, hot and cold compresses, frequent breastfeeding, cabbage leaf compresses, acupressure, and plants [4,7,12,17,18]. The current study was conducted to ascertain the effectiveness of alternating applications of cold and hot compresses for reducing breast engorgement among nursing women. The dearth of research on breast engorgement therapies in KSA motivated the study.

Interpretation of findings

Sociodemographic, Weight, Height, BMI, Past Medical and Surgical History, and Obstetric Data

The results showed that the majority of the participants were aged more than 25 and less than 35 and had high school-level, graduate-level, or postgraduate-level education. Furthermore, the majority were housewives and had one to three children. The majority of participants from both groups were overweight. Nearly half of the participants from both groups had no medical or surgical history. No statistical differences were found between both groups in relation to sociodemographic data (weight, height, and BMI).

The results also revealed that the majority of the intervention group had three to four antenatal visits, and the majority of the control group had more than five visits. The majority of both groups did not receive breastfeeding education or breast care education and experienced breast problems during pregnancy, with flat nipples being the most common problem. Additionally, the majority of the intervention and control groups did not undergo breast examinations performed by physicians. The majority of the intervention group and half of the control group reported milk production in their breasts. Furthermore, the majority of the intervention and control groups experienced discomfort during breastfeeding.

The results also showed that more than one-third of both groups were multipara, were four days postpartum, initiated breastfeeding within eight hours, and fed their infants for 10-20 minutes using the sitting position. The comparison between the intervention and control groups revealed that there were no statistically significant differences in approximately all the maternal obstetrical data.

The above findings are consistent with those of Monazzami et al. [4], who showed that the mean age in the intervention group was 28.76±6.23 years, whereas the mean age in the control group was 28.55±6.41 years. Most members of the control and intervention groups received diplomas. Additionally, the majority of them were obese. Participants’ age, educational attainments, family income, method of delivery, history of breastfeeding, number of births, and newborns’ weight did not significantly differ between the groups (P > 0.05).

Furthermore, in Khosravan et al. [1], the mean age of participants was 29.2±3.254 in the control group and 27.15±5.102 in the intervention group, which is consistent with the findings of the current study. There were no discernible variations between the two groups’ mean ages or BMIs, according to the Mann-Whitney test. The Chi-square test also revealed that the two groups were comparable in terms of education, parity, and method of delivery.

Additionally, in line with the findings of the current study, Eittah and Ashour’s [17] findings demonstrated that there were no statistically significant differences in terms of age and education level between the study participants (p ≥ .05). Napisah et al. [16] also revealed that the majority of respondents were in good health and of reproductive age, had high school education, were multiparous, and delivered their babies via cesarean section surgery. Additionally, their findings showed that there were no statistically significant differences in parity, nipple condition, or mode of delivery across the study participants.

Sharma’s [19] cesarean section results concurred with those of the current study. The majority of postnatal women are between the ages of 23 and 27 and come from rural communities, where they play the role of homemakers. The majority of postpartum women were literate up to the intermediate level. Some had graduate-level education. The majority of them had cesarean sections and witnessed symptoms of breast engorgement within one to three days of giving birth. Most mothers attempted to feed their children every two hours, up to 20 minutes each time.

In line with the findings of the current study, Aprillina et al. [20] found that postpartum women aged 20 to 35 who had only a junior high school degree, who were not employed or were homemakers, and who were multiparous mothers made up the majority of respondents who experienced breast engorgement.

Additionally, Zagloul et al. [7] showed that intervention group 1 and group 2 participants’ mean ages were 30.0±2.15 and 30.0±3.27 years, respectively. Regarding the start of feeding, their data showed that 16.6% and 20.0%, respectively, of group (A) and group (B) mothers had started nursing within the postpartum eight-hour period. Mothers fed their infants every time they wept for more than 30 minutes. No statistically significant sociodemographic or obstetric differences were found between the two groups.

The findings of Kumari [2], which supported the findings of the current study, showed that there were no statistically significant differences between the experimental and control groups in terms of sociodemographic and obstetric data. Additionally, the results of Lamadah et al. [8] showed that the mean age of the group receiving lukewarm water compresses was approximately 23 years and four months and that of the group receiving cold gel packs was approximately 25 years and six months. A total of 90% and 92.5%, respectively, of participants in both categories were homemakers. A significant percentage of participants in both groups (82.5% and 77.5%) were multiparas. Cesarean section delivery was reported in 67.5% and 55% of cases, respectively, but no statistically significant difference was discovered between the two groups. The findings also showed that breastfeeding started between eight and 24 hours following delivery.

Shamekh et al.’s [21] findings showed that the mean ages of the warm ginger and cold aloe vera gel compress groups, respectively, were 25.90±5.333 and 25.87±5.290 years. Regarding the occupation of each group’s participants, a sizable number (73.3% and 83.3%) of participants were homemakers. As expected, there were no statistically significant differences between the sociodemographic statistics of the two groups. The data also showed that both groups’ deliveries had one to three live children, with a mean parity of 2.30±1.00 and 2.17±0.96, respectively. Regarding the age of the participants, approximately 35 years and more of both groups, respectively, reported having experienced previous breastfeeding issues. Last, a large percentage of participants from the two groups experienced breast engorgement issues.

One possible reason the mean age of participants was 25-35 years in the present study’s results and the literature results are that this age range is the main reproductive age range. Further, the majority of participants were housewives. The majority of both groups were overweight, which may be due to carbohydrate-rich diets, a higher unemployment rate among women, social norms that frown on women exercising outside of the home, and finally, Arab men’s “preference” for curvy women. The justification for antenatal visits may be that health literacy is high today. The majority of both groups experienced discomfort during breastfeeding and four days postpartum. One of the inclusion criteria for the current study was that women must have signs of breast engorgement.

One possible reason participants initiated breastfeeding within eight hours and fed their infants for 10-20 minutes using the sitting position in both the current study and the literature is that the majority of them were multiparas and knew the importance of early breastfeeding. Additionally, one possible reason there were no significant differences between the intervention and control groups in relation to sociodemographic and obstetric data in the current study and the literature is that great control was maintained over the extraneous variables through various methods, such as random classification of both groups.

Breast Engorgement Level

The present study’s results showed that nearly half of the intervention group and nearly one-third of the control group had firm or beginning tenderness in the breasts pre-intervention, with no statistically significant differences between the two groups. After the intervention, none of the participants in the intervention group, as compared to 14.0% of the participants in the control group, had firm or beginning tenderness in the breast. There was a statistically significant difference between the two groups after alternating application of cold and hot compresses. The present study’s results showed no significant differences between the intervention and control groups before the alternating application of cold and hot compresses, whereas they did show significant differences between the intervention and control groups after the alternating application of cold and hot compresses.

According to Khosravan et al.’s [1] findings, which are consistent with the findings of the current study, the mean score for breast engorgement severity prior to the interventions was 10.05±2.438 for the control group and 9.15±2.412 for the intervention group, with the two groups being homogenous in terms of their pre-intervention scores (P = .234). Although there was a significant difference between the two groups, the generalized estimating equation showed that the intervention group (cold hollyhock leaf compresses combined with hot and cold compresses) had a mean overall score for breast engorgement that was significantly lower than that of the control group by a significant amount of 4.103 (P = .001).

The current findings were also consistent with those of Shamekh et al. [21], who found no statistically significant differences between the two groups. On the fifth day following the intervention, mild engorgement was found in 33.3% of participants in the warm ginger compress group compared to 16.7% of participants in the cold aloe vera gel compress group. On the seventh day following the intervention, no engorgement was found in 23.3% of the participants in the former group and 6.7% of the participants in the latter group. A highly statistically significant difference between each group before and after all therapies, with P = 0.0001, was also found.

Additionally, supporting the findings of the current investigation, Lamadah et al.’s [8] findings showed no statistically significant differences between the two groups before and after the first intervention day. After the second intervention day, a highly statistically significant difference (P = 0.000) was found. A statistically significant difference (P = 0.000) was also found in the lukewarm water compress group, showing that the amount of breast engorgement had decreased after the first and second intervention days.

In line with the findings of the current investigation, Zagloul et al. [7] found that both groups had significantly improved breast engorgement scores after the intervention (p = 0.001). The authors claimed that both interventions (hot compresses and cold cabbage) were successful at reducing breast engorgement and discomfort. Their findings also indicated that a number of interventions can reduce breast engorgement. The mean and standard deviation for groups A and B were respectively 3.743±0.205 and 4.26±0.171 after four interventions.

According to Aprillina et al.’s [20] findings, which were consistent with those of the current study, the majority of the 29 women participants (96.7%) reported experiencing engorgement on a scale of 3 prior to application of cold cabbage leaf compresses. This decreased to a scale of 2 following the intervention. Cold cabbage leaf compresses were effective at reducing postpartum women’s breast engorgement, with a value of P = 0.000.

Sharma’s [19] findings showed that both interventions (applying cold or hot cabbage leaves) were effective at reducing breast engorgement manifestations. This reduction was significant when the mean breast engorgement score was compared between the pretest and posttest in the experimental and control groups.

According to Thomas et al. [13], whose findings supported those of the current study, there was no significant difference between the two groups’ posttest breast engorgement scores (P = 0.204). Hot compresses and cold cabbage leaves were both beneficial for reducing breast engorgement in postpartum women (P = 0.05 and P = 0.001, respectively).

Gresh et al.’s [22] case study further emphasized the significance of healthcare professionals supporting evidence-based breastfeeding techniques and ensuring that breast engorgement is treated appropriately. They concluded that the use of hot and cold compresses following feedings was the most successful method for managing breast engorgement.

The findings of Monazzami et al. [4] showed that although the effects of breast engorgement severity were significantly greater in the hot compression group than in the control group (P = 0.001), the mean of total engorgement after intervention in the right and left breasts decreased in both groups.

According to Salgaonkar’s [23] review of the literature, the most common nonpharmacological techniques used to activate the milk ejection reflex and reduce breast engorgement are cold cabbage compresses, cold gel packs, hot compresses, and warm showers.

In contrast to the findings of the current study, Kaur et al.’s [24] findings showed that hot compresses worked better at reducing breast engorgement than cold ones do. Additionally, Kumari’s [2] findings demonstrated that the hot water bag group experienced a drop in its engorgement score from 6 to 4, whereas the cold green cabbage leaf group experienced a drop in its engorgement score from 6 to 3. The level of engorgement decreased more quickly in the cold cabbage leaf group than in the hot water bag group.

A possible reason for both the present study’s results and the literature’s results showing that hot or cold compresses alone or both of them together with or without additives such as cabbage leaves or ginger can reduce breast engorgement levels is that plugged milk is the main cause of breast engorgement. It leads to breast swelling and tenderness. Applying alternate hot and cold compresses helps to reduce the level of engorgement. Cold compresses help to reduce swelling, whereas hot compresses help to increase milk flow.

Meanwhile, a possible reason for the contradiction between the present study’s results and the literature results regarding hot and cold compresses, with the latter shown to be more effective in some studies and the former shown to be more effective in other studies, might be that the bulk of those studies analyzed the effects of the two approaches separately. They discovered that while both methods had an impact on breast engorgement, one method had a stronger impact than the other. In contrast, the current study compared the effects of alternating the two methods.

Pain Level

The present study revealed that none of the participants in the intervention group, as compared to some of the participants in the control group, had any pain preintervention. A statistically significant difference was found between the two groups before the intervention. However, after the intervention, the majority of the intervention group, as compared to nearly one-quarter of the control group, had no pain. A statistically significant difference was found between the two groups after the intervention.

The present study’s findings supported those of Kumari [2], who showed that the mean initial pain score for both groups from baseline to after 20 minutes was the same and that both groups’ pain scores steadily decreased over the course of six time intervals. There was an equal decrease in the hot water bag group and the cold green cabbage leaf group.

According to Eittah and Ashour’s [17] results, which concurred with those of the current study, the mean pain scores for both groups improved after the use of hot compresses and cold cabbage leaves; the improvement was highly statistically significant. Consistent improvement was seen between the second and sixth applications.

Zagloul et al. [7], confirming the findings of the current study, showed that postintervention pain scores significantly improved (P = 0.001). Further, following the intervention, there was an average improvement of 0.51±0.4 and 2.97±0.2 in group (A) and 3.02±0.2 and 3.45±0.4 in group (B), respectively. They concluded that both methods of pain relief (hot compress and cold cabbage) worked well.

Next, supporting the findings of the current study, Ketsuwan et al. [12] discovered that there were statistically significant mean differences in breast-engorgement-related pain between the herbal and hot compress groups before and after therapy. Both treatments lowered pain levels according to the study findings: hot compresses alone reduced pain scores from 5.8 to 2.8 (on a scale of 10; P = .001), and hot compresses reduced reported pain scores from 6.9 to 1.0 (on a scale of 10; P = .001).

In agreement with the present study’s results, Monazzami et al.’s [4] Mann-Whitney U-test results revealed no significant between-group differences with regard to the pretest mean scores for breast engorgement-associated pain (P > 0.05). After the intervention, their Wilcoxon test intragroup comparisons showed that the mean scores for breast-engorgement-related pain in the right and left breasts significantly decreased by 6.25±1.76 and 6.06±1.76 points, respectively, in the intervention group (P = 0.05). The mean scores for breast-engorgement-related pain in the right and left breasts significantly decreased in the control group by 3.21±1.02 and 3.48±1.21 points, respectively (P = 0.05).

A possible reason for the present study’s results and the literature results that hot or cold compresses alone or both of them together with or without additives such as cabbage leaves or ginger can reduce pain is that using cold compresses can considerably reduce inflammation and swelling, which produce pain by reducing blood supply to the breasts through vasoconstriction. It can also diminish nerve activity, which aids in pain reeducation. Additionally, hot compresses help to open up the milk ducts, thus softening the breast. This reduces inflammation and makes it easier for milk to flow from the breast. Heat also has analgesic effects. By stimulating the skin’s sensory receptors and reducing the number of pain signals reaching the brain, thus reducing swelling and speeding up the process of relieving pain, using both methods alternately can be more beneficial than using one method exclusively.

LATCH on Level

Regarding LATCH, there were no significant differences between the intervention and control groups before the intervention, whereas there were significant differences between the groups after the intervention.

The findings were consistent with those of Rahnemaie et al. [25], who showed that complementary medicine practices are helpful in resolving breastfeeding issues and in empowering mothers to undertake successful breastfeeding. These in turn result in reducing pain and improving latch.

Additionally, Hassan et al.’s [11] results showed that the cold cabbage leaf groups experienced complete recovery from pain, redness, hardness, and pyrexia, with an increasing LATCH score during the four postpartum visits. In the fenugreek hot seed group, full recovery from pain, pyrexia, and an increasing LATCH score occurred within 24 hours of the intervention, whereas full recovery from redness and hardness occurred 36 hours later. The authors concluded that, regardless of the employed metric, a significant improvement in breast condition and LATCH was observed following the intervention for both groups.

El-Hady et al.’s [9] results, which were consistent with the results of the current study, showed the distribution of the examined primipara postnatal women according to the overall level of the LATCH Breastfeeding Charting Scale. After administering self-care techniques including hot and cold compresses, 63.5% of women were found to be fair breastfeeders, 27.0% were found to be good breastfeeders, and 9.5% were found to be bad breastfeeders.

Mahdizadeh-Shahri et al. [26] noted that the mean scores for all breastfeeding success factors, including readiness to feed, fixing (latching on), and sucking, in the breastfeeding mothers in the intervention group in both stages were significantly higher than those for the participants in the control group (P = .001). This finding lends credence to the findings of the current study. According to the LATCH scale results, the mothers in the intervention groups required less assistance than the participants in the control group (P = .001).

Ebrahim and Esmat [27] demonstrated that there was no statistically significant difference in terms of pain intensity, breast engorgement, or LATCH scale scores before the implementation of an educational program between the warm and cold cabbage groups. However, after the implementation of the program, there was a highly statistically significant improvement in LATCH scale scores and a highly statistically significant reduction in the degree of pain and breast engorgement between the groups, particularly the hot compress group.

According to Farag et al.’s [28] results, which concurred with the findings of the current study, 37.5% of the control group practiced inadequate breastfeeding before the intervention, compared to 45% of the intervention group. Following post assessment 2, these percentages decreased to 22.5% in the control group and 0% in the intervention group. Before the intervention, the control and intervention groups reported good breastfeeding rates of about 22.5% and 25%, respectively. After post assessment 2, there was a highly statistically significant difference between the control and intervention groups, with P = 0.001. This percentage changed to 27.5% in the control group and 50% in the intervention group.

A possible explanation for the present study’s results and the literature’s results that cold and hot compresses are beneficial in increasing the latch level may be that moist hot compresses give warmth to a targeted area of the body. Heat application activates the skin’s nerves and quickly raises its warmth. The amount of blood circulating to that location rises as a result. Wider blood vessels help carry more nutrients and white blood cells to the location by increasing blood flow. Relaxing muscles, releasing oxytocin, and ejecting milk are just a few of the actions that can assist the body. These actions lead to better breast conditions, which in turn improve latch quality. Additionally, applying a cold compress reduces swelling and fullness by reducing fluid in the alveolar tissue.

Breastfeeding Assessment and Mother Evaluation of Breastfeeding

The present study showed that before the intervention, there was no statistically significant difference between the intervention and control groups in relation to all items of breastfeeding assessment (baby arousal, rooting, duration between latch and suck, and sucking pattern) and total levels of infant breastfeeding assessment over the study period. After the intervention, there was a statistically significant difference between the intervention and control groups in relation to all items of breastfeeding assessment and total levels of infant breastfeeding assessment over the study period. Further, the present study found that nearly half of the mothers in the intervention group, as compared to only 8.0% of the mothers in the control group, were pleased with their breastfeeding. A statistically significant difference existed between the intervention and control groups in relation to mothers’ evaluation of their breastfeeding.

According to Monazzami et al.’s [4] findings, which are consistent with the findings of the current investigation, ginger compress and hot compress can be used to improve the quality of postpartum care and breastfeeding outcomes and support women in breastfeeding.

In line with the findings of the current study, Wong et al.’s [3] findings indicated that the cold cabbage leaf and gel pack group showed statistically significant improvement postintervention compared to preintervention in terms of overall levels of baby breastfeeding assessment. Mothers liked the breast engorgement care given in the cold cabbage leaf and gel pack groups more.

Additionally, Wahyuningsih and Liliana’s [29] findings showed that the control group did not differ significantly from the hot compress group in terms of breast milk production. However, there was a significant difference in terms of breastfeeding assessment criteria between the groups before and after the intervention. The difference in breast milk production between the intervention group and the control group was statistically significant.

In line with the current study’s findings, Wang and Li’s findings showed that there was no significant difference in postpartum breastfeeding status, comfort nursing score, or milk production between the two groups prior to intervention [30]. Following the intervention, the intervention group’s breastfeeding status, comfort nursing score, and milk production were higher than the control groups. The intervention group’s satisfaction with breastfeeding was higher than that of the control group as well.

In line with the findings of the current study, Rahnemaie et al.’s [25] findings showed that complementary medicine practices, such as the use of herbal supplements, massage/point massage, and hot and cold compress, can be used as low-risk, safe, and affordable ways to promote exclusive breastfeeding, assess the amount of breastfeeding, and promote general well-being.

Mahdizadeh-Shahri et al. [26], supporting the findings of the current study, noted that control group participants’ mean scores for readiness to feed, rooting, fixing, and sucking were significantly lower than those for the intervention group participants at both stages (P = .001). Regarding the requirement for breastfeeding support in both breastfeeding rounds, the study’s findings revealed a considerable difference between the two groups.

A possible reason the present study’s results and the literature results showed an improvement in all items of breastfeeding assessment and total levels of breastfeeding assessment and the mother’s satisfaction with breastfeeding is that warm breast compresses promote breast milk ejection from the lactiferous ducts and relieve breast engorgement in postpartum women. Additionally, the physiological effects of applying hot compresses include vasodilation, increased capillary permeability, muscle relaxation, and increased blood flow to the breast area. This increased blood circulation in the breast area causes more oxytocin to flow to the breast, facilitating breastfeeding. The improvement in breast health and the rise in milk production enhance maternal happiness and breastfeeding indicators, including baby alertness, rooting, length between latch and suck, and sucking rhythm. Additionally, using a cold compress helps mitigate discomfort during feeding by reducing pain and swelling. This can enhance breastfeeding indicators including baby arousal, rooting, length between latch and suck, and sucking rhythm as well as the mother’s satisfaction.

Relations

The current study revealed that there was no significant relation between participants’ obstetrical data and their breast engorgement except in regard to the number of antenatal visits, frequency of feeding, mother’s position during breastfeeding, discomfort during breastfeeding, and number of postpartum days. There was no significant relation between participants’ obstetrical data and level of pain except in regard to discomfort during breastfeeding, immediate skin-to-skin contact, breast problems during pregnancy, mother’s position during breastfeeding, bottle feeding, number of postpartum days, and frequency of feeding per hour. There was no significant relation between participants’ obstetrical data and their level of latch except in regard to breast examination by a physician, milk production, immediate skin-to-skin contact, duration of feeding during each breastfeeding, breast problems during pregnancy, discomfort during breastfeeding, parity, and number of postpartum days.

Supporting the findings of the present study, Zagloul et al. [7] indicated that the frequency of feeding, the mother’s position while breastfeeding, and discomfort during breastfeeding affected breast engorgement and pain. The mean and standard deviation for groups A and B, respectively, were 3.743±0.205 and 4.26±0.171. The statistical differences between the two groups were significant (P = 0.012*). Milk production, quick skin-to-skin contact, and feeding time also influenced latch level. The mean and standard deviation for groups A and B were 3.852±0.202 and 4.15±0.154, respectively. The differences between the two groups were significant (P = 0.001*).

Supporting the present study’s findings, Khosravan et al. [1] showed that there was no significant relationship between the scores for breast engorgement severity and pain level and the majority of obstetric data, but there was a significant relationship between these variables and the time or duration of breastfeeding, the presence of breast problems, and the number of postpartum days (P = .001). The negative coefficient denoted a declining mean score over time.

In agreement with the present study’s findings, El-Hady et al. [9] found that there were significant associations between breast engorgement and women’s frequency of feeding and discomfort during feeding (P = 0.007* and 0.001*, respectively). They also showed that there were significant correlations between the total LATCH scale scores and the following variables: the number of postpartum days (P = 0.0001*), the amount of milk produced (P = 0.0001*), the number of breast problems (P = 0.016*), and the frequency of feeding (P = 0.039*).

A possible reason for the present study’s results and the literature results showing that there is a significant relation between engorgement and the number of antenatal visits is that the goal of antenatal visits is to prepare the mother for the minor discomforts of pregnancy and the postpartum period. The number of postpartum days may be due to the fact that mothers in early postpartum experience more engorgement than mothers in late postpartum as a result of milk volume and feeding amount. The frequency of feeding also decreases the level of engorgement and congestion. Last, the mother’s position during breastfeeding helps to decrease engorgement because the sitting position is considered to be the least uncomfortable.

A possible reason for the present study’s result that there is a significant relation between the level of pain and discomfort during breastfeeding may be that if women feel discomfort, by default, they will experience an increase in pain level. The mother’s position during breastfeeding may affect the pain level. The sitting position enhances drainage and reduces engorgement. Immediate skin-to-skin contact may also affect the pain level because it increases the maternal-fetal bond. A mother will experience less pain if she has a stronger bond with her child. Breast problems during pregnancy may also affect the pain level because they may be related to the mother’s prior experience with breast pain. Mothers in the early postpartum period experience more engorgement and more pain than in the later period because of the effects of the milk volume and the feeding amount. The frequency of feeding also decreases the level of engorgement and increases the pain level. The number of postpartum days and the frequency of feeding per hour are also affected by the pain level.

A possible explanation for the present study’s results and the literature results showing that there is a significant relation among level of latch, breast examination by physician, milk production, immediate skin-to-skin contact, duration of feeding, breast problems during pregnancy, discomfort during breastfeeding, parity, and number of postpartum days is that the engorgement and pain level can affect and be affected by the earlier aspects. If the effect is favorable and the engorgement and pain level improve better latch indicators will result, and vice versa.

According to the previously discussed studies, breast engorgement and soreness are uncomfortable and can result in consequences like breast inflammation, sore or cracked nipples, and decreased milk production. Women might quit breastfeeding as a result. The evidence supporting treatments that work consistently is contradictory, although the majority of them endorse non-pharmacological approaches and attest to their effectiveness. There is some evidence that treatments like hot and cold compresses, cabbage leaves, cold gel packs, herbal compresses, and massage, may be effective at treating breast engorgement. However, the main finding is that all of these methods work to reduce the pain associated with engorgement.

Limitation

The primary limitation of the current study is as follows: the small sample size of participants makes it difficult to generalize the results. Additionally, because the study was solely focused on Al-Ahsa city, the results cannot be generalized to other areas with distinct features. The following variables contributed to the small sample size: specific requirements for the inclusion of participants, and some mothers decided not to participate in the study, whereas others did not want to finish it.

Strengths of the study

The present study is one of the few nursing studies conducted in KSA to assess the effectiveness of the application of alternate cold and hot compresses to reduce breast engorgement in lactating women. This technique is beneficial for reducing patients’ discomfort and pain related to milk stasis. Furthermore, it is efficient and easy to perform. Education regarding complementary therapies for breast engorgement management, including safe and simple treatment with hot compress and cold gel packs, should be given to healthcare professionals, especially nurses and midwives.

## Conclusions

According to the results of this study, applying cold gel packs and hot compresses to the breasts can reduce breast engorgement. Additionally, there was a statistically significant variation between the level of pain score and engorgement scores for both groups pre and post-test (P = 0.005*). The alternating of hot and cold compresses can decrease the breast pain from severe to moderate and from moderate to mild in addition to lowering the breast engorgement score. The results presented could help Saudi Arabian nurses and midwives better understand the advantages of alternate applications of cold gel packs and hot compresses, which lower the likelihood that a mother will develop breast engorgement and cease breastfeeding early.

The researcher recommended future clinical practice where women should be encouraged to use hot compresses and cold gel packs as an alternative treatment to reduce engorgement and promote comfort. In addition, utilize the study results to aid Saudi Arabian nurses, midwives, and postpartum mothers in understanding the advantages of applying a cold gel pack and a hot compress, to decrease levels of engorgement, improve latch, and relieve discomfort. This method is a simple, inexpensive, and safe treatment that is easy to use. Furthermore, providing programs for continuing education for maternity nurses on the use of several techniques and approaches is recommended to reduce breast engorgement and pain. Also, distribution of posters, booklets, and other types of educational materials to postpartum mothers and nurses to inform them of the most crucial educational advice that should be used to promote successful breastfeeding without complications.
